# Molnupiravir combined with different repurposed drugs further inhibits SARS-CoV-2 infection in human nasal epithelium *in vitro*

**DOI:** 10.1016/j.biopha.2022.113058

**Published:** 2022-06

**Authors:** Hulda R. Jonsdottir, Denise Siegrist, Thomas Julien, Blandine Padey, Mendy Bouveret, Olivier Terrier, Andres Pizzorno, Song Huang, Kirandeep Samby, Timothy N.C. Wells, Bernadett Boda, Manuel Rosa-Calatrava, Olivier B. Engler, Samuel Constant

**Affiliations:** a Spiez Laboratory, Federal Office for Civil Protection, Spiez, Switzerland; b Department of Rheumatology, Immunology, and Allergology, Inselspital University Hospital, Bern, Switzerland; c Department of BioMedical Research, University of Bern, Bern, Switzerland; d VirNext, Faculté de Médecine RTH Laennec, Université Claude Bernard Lyon 1, Université de Lyon, 69008, Lyon, France; e CIRI, Centre International de Recherche en Infectiologie, (Team VirPath), Univ Lyon, Inserm, U1111, Université Claude Bernard Lyon 1, CNRS, UMR5308, ENS de Lyon, F-69007 Lyon, France; f Epithelix Sàrl, Plan-les-Ouates, Switzerland; g Medicines for Malaria Venture, Geneva, Switzerland

**Keywords:** SARS-CoV-2, COVID-19, Air-liquid interface, Drug repurposing, Antivirals

## Abstract

The severe acute respiratory syndrome coronavirus 2 (SARS-CoV-2) has caused a worldwide pandemic with unprecedented economic and societal impact. Currently, several vaccines are available and multitudes of antiviral treatments have been proposed and tested. Although many of the vaccines show clinical efficacy, they are not equally accessible worldwide. Additionally, due to the continuous emergence of new variants and generally short duration of immunity, the development of effective antiviral treatments remains of the utmost importance. Since the emergence of SARS-CoV-2, substantial efforts have been undertaken to repurpose existing drugs for accelerated clinical testing and emergency use authorizations. However, drug-repurposing studies using cellular assays often identify hits that later prove ineffective clinically, highlighting the need for more complex screening models. To this end, we evaluated the activity of single compounds that have either been tested clinically or already undergone extensive preclinical profiling, using a standardized *in vitro* model of human nasal epithelium. Furthermore, we also evaluated drug combinations based on a sub-maximal concentration of molnupiravir. We report the antiviral activity of 95 single compounds and 30 combinations. We show that only a few single agents are highly effective in inhibiting SARS-CoV-2 replication while selected drug combinations containing 10 µM molnupiravir boosted antiviral activity compared to single compound treatment. These data indicate that molnupiravir-based combinations are worthy of further consideration as potential treatment strategies against coronavirus disease 2019 (COVID-19).

## Highlights

• Not many single compounds exhibit antiviral activity against SARS-CoV-2.
• The RdRp inhibitor, molnupiravir, inhibits SARS-CoV-2 at various concentrations.
• Combinations containing 10 µM molnupiravir boost antiviral activity.
• Combination treatment might provide additional therapeutic benefit against COVID-19.

## Graphical Abstract

**Figure:**
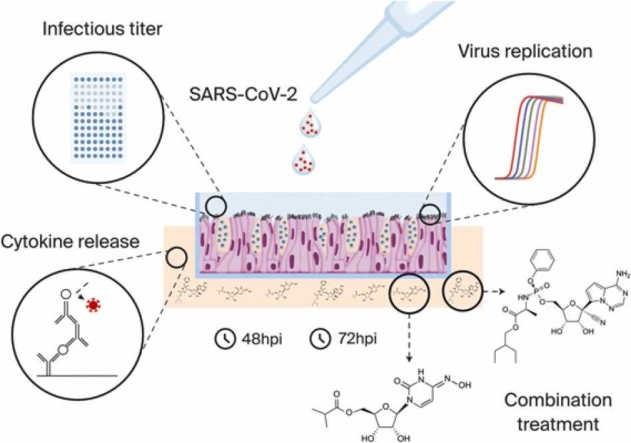

## 1 Introduction

In December 2019, a novel coronavirus (CoV) causing respiratory illness in humans was identified in Wuhan, China [1]. In the following months, this virus, designated severe acute respiratory syndrome coronavirus 2 (SARS-CoV-2) [2], spread to every continent and, as of 28 March 2022, there have been almost 480 million documented cases worldwide and over 6 million deaths [3]. The corresponding illness, coronavirus disease 2019 (COVID-19), presents with a range of symptoms and severity [4], [5], [6], [7], [8]. The acute respiratory disease syndrome (ARDS) observed in severe cases of COVID-19 is driven by a cytokine storm, characterized by the uncontrolled overproduction of soluble immune mediators. This results in sustained inflammation and tissue injury and can lead to low oxygenation followed by death [9], [10]. A recurring pattern of upregulation of pro-inflammatory cytokines, including interleukin (IL)− 6, IL-1β, and tumor necrosis factor-α, has been observed [11], [12] with the additional secretion of various chemokines, *e.g.*, monocyte chemoattractant protein-1, interferon gamma-induced protein (IP)− 10, and RANTES resulting in the influx of immune cells into the pulmonary space [13], [14], [15], [16]. Despite the rapid development and current availability of several vaccines, effective and well-tolerated antiviral treatments against COVID-19 are still of extreme importance due to incomplete vaccine uptake, incomplete protective response, and the relatively short duration of immunity. Additionally, those who cannot be vaccinated or choose not to be, are not protected against severe COVID-19. Development of new treatments traditionally takes many years; however, the rapid spread of the virus means that new treatment options against SARS-CoV-2 are urgently needed. Drug repurposing redirects existing or previously approved drugs as new therapeutics for new clinical indications in a time- and cost-effective manner. For example, remdesivir, also known as GS-5734, an intravenous ProTide prodrug [17] targeting the RNA-dependent RNA polymerase (RdRp) of Hepatitis C virus (HCV), was repurposed and conditionally approved as an emergency treatment for COVID-19 early in the pandemic [18]. However, due to the complex interplay between the host immune system and virus replication, drugs inhibiting viral replication alone are often insufficient to fight the systemic viral infection and resulting sequelae. For early ambulatory treatment of COVID-19, various treatments combining compounds targeting viral replication with other drugs have been proposed to maximize therapeutic potential [19], [20], [21]. In addition, experiences gained from the development of therapeutic strategies against other RNA viruses, *e.g.*, human immunodeficiency virus (HIV) and HCV, underline the importance of combining several drugs targeting different viral proteins or host factors to prevent the emergence of drug-resistant mutants [22], [23], [24]. Due to the heterogeneity of *in vitro* cell lines used in screening studies, direct comparison of drug efficacy can be difficult. We developed a standardized *in vitro* screening model using fully differentiated primary human nasal epithelium (MucilAir™) to facilitate a more direct comparison of compound efficacy. Applying a standardized test protocol, we re-evaluated a collection of compounds from the Medicines for Malaria Venture (MVV) open-access collections [25] and the Calibr ReFRAME drug-repurposing library [26]. In the current study, we assessed the antiviral potential of 99 single compounds at two study sites focusing on orally active drugs. Subsequently, we selected molnupiravir, an orally bioactive RdRp inhibitor recently approved by the American Food and Drug Administration (FDA), as a base compound for combination therapy [27]. We evaluated various concentrations of 30 drugs in combination with 10 µM molnupiravir against SARS-CoV-2 infection in reconstituted nasal epithelium. We found that selected combinations have the potential to boost antiviral activity compared to single treatments, indicating a benefit of combined antiviral treatment against SARS-CoV-2.

## 2 Materials and methods

### 2.1 Reconstituted nasal epithelium - MucilAir™

Human nasal epithelial cells were obtained from patients undergoing polypectomy. Experimental procedures were explained in full, and all subjects provided informed consent. The study was conducted according to the declaration of Helsinki (Hong Kong amendment, 1989), and received approval from local ethics commissions. Cells were isolated from primary tissue as previously described [28] and expanded once (p1). Pooled nasal epithelial cells from 14 individual donors were then seeded on 6.5-mm Transwell® inserts (cat #3470, Corning Incorporated, Oxyphen, Wetzikon, Switzerland) in MucilAir™ culture medium (EP04MM, Epithelix Sàrl, Geneva, Switzerland). Once confluent, air-liquid interface (ALI) was established and maintained for at least 28 days for mucociliary differentiation. Average culture time post-ALI establishment was 43 days.

### 2.2 Compound selection and toxicity

Compounds were sourced from Calibr (California Institute for Biomedical Research, La Jolla, California, USA) or from MMV except for IFN-α (IF007 #3308269), IFN-λ (SRP3060), ebselen (E3520), dalbavancin (SML-2378) and nanchangmycin (SML2251 #0000040357) which were purchased from Sigma Aldrich (Buchs SG, Switzerland), while IFN-β (AF-300–02B) was sourced from Peprotech (LubioScience GmbH, Zürich, Switzerland). Compounds were selected using available data from primary screens using various cell lines (Vero E6, HeLa-ACE2, Calu-3) and organoids and were obtained blinded as 10 mM stock solution in dimethyl sulfoxide (DMSO). Compound toxicity (n = 104) was evaluated by assessing the release of lactate dehydrogenase (LDH, Assay Kit-WST CK12–20, Dojindo EU, Munich, Germany) from damaged cells at 48 and 72 h post-treatment. Compound-containing cell culture media was replenished every 24 h. Trans-epithelial electrical resistance (TEER) was monitored at 24, 48, and 72 h, while ciliary beating frequency (CBF) was assessed at 72 h by capturing 256 images at a high-frequency rate (125 frames per second) at room temperature (RT). CBF was calculated using Cilia-X software (Epithelix Sàrl, Geneva, Switzerland).

### 2.3 Determination of epithelial integrity

Epithelial integrity was determined by quantifying TEER at 48 and 72 h post-infection (hpi) using the EVOM3/EVOMX Volt/Ohm Meter and the accompanying chopstick electrode (STX2/STX2-plus, World Precision Instruments, Friedberg, Germany). 200 µl OptiMEM (Gibco, Thermo Fisher, Basel, Switzerland) or 0.9% NaCl solution was added to the apical compartment prior to measurement. Total resistance values (Ω) were converted to TEER (Ω cm^−2^) by subtracting 100 Ω for the resistance of the polyester membrane and multiplying by 0.33 cm^2^.

### 2.4 Virus propagation

SARS-CoV-2 (BetaCoV/France/IDF0571/2020, EPI_ISL_406596), used at the primary testing site, was acquired from EVAg (Emerging Viral Diseases Medical Faculty, Marseille, France) and propagated on Vero E6 cells (provided by Prof. Dr. Volker Thiel, University of Bern, Bern, Switzerland) in Minimum Essential Medium (MEM; Seraglob, Schaffhausen, Switzerland) supplemented with 2% Fetal Bovine Serum (FBS; Seraglob) at 37 °C, 5% CO2, and > 85% relative humidity (rH) for 3 days prior to harvest. The SARS-CoV-2 strain used at the second testing site was isolated from a patient in a French clinical cohort of patients with COVID-19 (NCT04262921) in January 2020 at the Department of Infectious and Tropical Diseases, Bichat Claude Bernard Hospital, Paris (BetaCoV/France/IDF0571/2020, EPI_ISL_411218) [29]. Tissue culture infectious dose 50 (TCID50/ml) was determined by a limiting dilution assay under the same culture conditions with the Spearman-Kärber method [30], [31] or the Reed & Muench statistical method [32], [33], depending on the testing site.

### 2.5 Virus infection

**Fig. 1:**
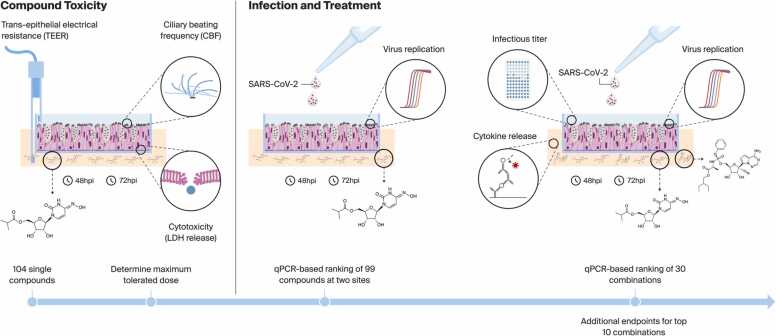
Overview over experimental design and layout. Toxicity of 104 single compounds was determined in reconstituted nasal epithelium *in vitro* to determine the maximum tolerated concentration (≤30 µM). Compounds were applied basally, mimicking systemic administration. Three endpoints were assessed, trans-epithelial electrical resistance (TEER), cytotoxicity, and the ciliary beating frequency of ciliated cells. Subsequently, 99 single compounds were screened for antiviral activity against SARS-CoV-2. Additionally, 30 combinations based on the orally bioactive RdRp inhibitor molnupiravir were tested for antiviral activity with additional parameters (infectious virus and cytokine secretion) determined for top candidates.

Prior to infection, duplicates of MucilAir™ reconstituted nasal epithelium were washed twice with OptiMEM warmed to 37 °C and basal media replenished with warm MucilAir™ cell culture media. 5 × 10^4^ TCID_50_ of SARS-CoV-2 (for a theoretical MOI of 0.1) were added to the apical compartment diluted in OptiMEM and incubated at 37 °C, 5% CO_2_, and > 85% rH for 1 h. Mock controls were exposed to the same volume of OptiMEM only. Subsequently, virus inoculum was removed, and the epithelium transferred to cell culture media containing test or control compounds. Apical virus release was assessed at 48 and 72 hpi by washing the apical side with 200 µl OptiMEM for 10 min at 37 °C. During the harvest of viral particles from the apical compartment, *i.e*., while the epithelium is submerged, TEER was assessed as described in chapter 2.3. Test and control compounds were replenished every 24 h for the duration of the experiment (72 h). Remdesivir was applied as a positive control at a concentration of 5 μM (Gilead Sciences Inc., Foster City, California, USA or MedChemExpress, HY-104077). All compounds were diluted in DMSO and final concentration of DMSO in the antiviral assay was standardized as 0.3% for single compounds and 0.4% for combinations. Overview of experimental layout is presented in Fig. 1.

### 2.6 RNA extraction

200 µl of apical wash was harvested at 48 and 72 hpi and 100 µl inactivated in 400 µl AVL buffer (Qiagen, Hombrechtikon, Switzerland) for 10 min at RT followed by the addition of 400 µl absolute ethanol to each sample. Viral RNA was extracted from 500 µl with the MagNaPure 96 system (Roche, Basel, Switzerland) according to the manufacturer’s instructions and extracted RNA eluted in 100 µl. Alternatively, 140 µl were used for viral RNA extraction with the QIAamp® Viral RNA kit (Qiagen, Hombrechtikon, Switzerland), obtaining 60 µl of eluted RNA. At the end of each experiment, epithelia were lysed in Qiazol (Qiagen, Hombrechtikon, Switzerland) for a total of 20 min at RT. RNA was extracted using the Qiagen RNeasy Plus Universal Mini kit according to the manufacturer’s instruction, eluted in 30 µl of nuclease-free water, and diluted 1:10 prior to gene expression analysis.

### 2.7 qRT-PCR

Viral RNA in apical secretions was quantified using the TaqMan™ Fast-Virus-1 Step master mix (Thermo Fisher, Basel, Switzerland) with these cycling parameters: 50 °C for 1 min, 95 °C for 20 s, 45 cycles of 95 °C for 3 s and 60 °C for 30 s using the LightCycler 96 system (Roche, Basel, Switzerland) or the StepOnePlus™ Real-Time PCR System (Applied Biosystems, Massachusetts, USA) with the following primers against SARS-CoV-2 non-structural protein 14 (nsp14): Fwd: 5′-TGGGGYTTTACRGGTAACCT-3′, Rev: 5′-AACRCGCTTAACAAAGCACTC-3′. Probe: 5′-FAM-TAGTTGTGATGCWATCATGACTAG-TAMRA-3′ (protocol by Leo Poon, Daniel Chu, and Malik Peiris; School of Public Health, The University of Hong Kong, Hong Kong). Changes in gene expression were calculated using the 2^-ΔCt^ method and reported as the fold reduction over infected vehicle-treated inserts (virus control, 0.3–0.4% DMSO). Intracellular RNA was quantified using the SuperScript III Platinum One-step SYBR Green master mix (Thermo Fisher, Basel, Switzerland) and these cycling parameters: 50 °C for 1 min, 95 °C for 5 min, 50 cycles of 95 °C for 15 s, 60 °C for 30 s (53 °C for nsp14), 40 °C for 1 min followed by a melting curve to confirm product specificity or the StepOnePlus™ Real-Time PCR System (Applied Biosystems) using the previously mentioned primers against nsp14 and the following primers against GAPDH: Fwd: 5′-GAAGGTGAAGGTCGGAGTCAAC-3′, Rev: 5′-CAGAGTTAAAAGCAGCCCTGGT-3′ [34]. Changes in intracellular gene expression were calculated using the 2^-ΔΔCt^ method and reported as the fold reduction over infected vehicle-treated inserts (virus control, 0.3–0.4% DMSO). Ct values > 40 were considered not-detected. In the current study, this corresponds to a maximum log_10_ fold change of − 7.

### 2.8 Determination of infectious titer

To quantify infectious virus in apical secretions, apical wash samples (72 hpi) were diluted in MEM supplemented with 2% FBS and titrated as described in chapter 2.4. Vero E6 cells modified to constitutively express the serine protease TMPRSS2 (RRID:CVCL_YQ49) [35] were obtained from the Center for AIDS Reagents (National Institute for Biological Standards and Control) and were incubated for 3 days at 37 °C, 5% CO_2_, and > 85% rH prior to determination of cytopathic effect by crystal violet staining. Infectious titer was determined with the Spearman-Kärber method [30], [31], [33] and reported as tissue culture infectious dose 50 per ml (TCID_50_/ml).

### 2.9 Determination of cytokine release

Secretion of CXC motif chemokine ligand 10 (CXCL10/IP-10, DY266) and CC chemokine ligand 5 (CCL5/RANTES, DY278) were quantified by DuoSet® ELISA (R&D systems, Bio-Techne GmbH, Wiesbaden, Germany) under BSL-3 conditions according to the manufacturer’s instructions using the appropriate ancillary reagent kit (DY007).

### 2.10 Statistical analysis

Statistical significance between single molnupiravir treatments and combinations were determined by ordinary one-way analysis of variance (ANOVA) with Dunnett’s multiple comparison test. Significance between single and combination treatment for other compounds was determined by ordinary one-way ANOVA with Sidak’s multiple comparison test. p-values < 0.05 were considered statistically significant. All statistical analyses were performed with GraphPad Prism version 8.4.3 for Mac OS X, GraphPad Software, San Diego, California, USA, www.graphpad.com.

## 3 Results

### 3.1 Compound toxicity

Tolerated concentrations of all compounds were evaluated up to 30 µM on pooled nasal epithelium (MucilAir™, data not shown). The maximum tolerated concentration (MTC ≤30 µM) for each of the tested compounds is reported in Table S1. Examples of a well-tolerated compound, molnupiravir, and a compound inducing toxic effects, LY2228820, are shown in Fig. S1. Compounds that resulted in > 5% cytotoxicity as evaluated by the release of lactate dehydrogenase (LDH) and severe loss of epithelial integrity (TEER < 100 Ω cm^−2^) after three days of treatment were considered toxic. Reduced ciliary beating frequency (CBF) served as further confirmation of compound toxicity. Only non-toxic compounds were tested for antiviral activity. However, we also observed that treatment with some of the non-toxic single compounds including osimertinib, midostaurine, ZLVG CHN2 and ozanimod, led to a loss of epithelial integrity, beyond that observed for vehicle control, only after infection with SARS-CoV-2. Due to ongoing compound shortages, the toxicity of compound combinations was tested indirectly during SARS-CoV-2 infection by monitoring TEER. All toxic compounds and combinations were excluded from further analysis (data not shown).

### 3.2 Antiviral activity of single compounds against SARS-CoV-2

**Fig. 2:**
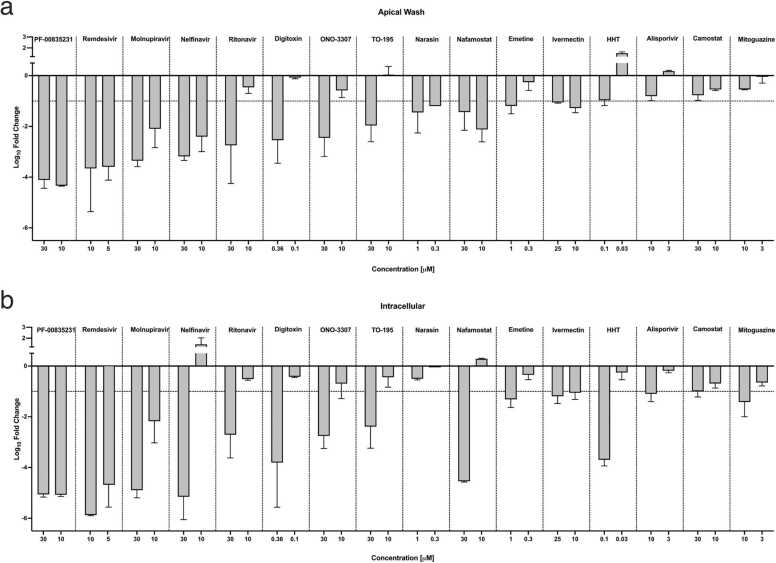
Antiviral activity of single compounds against SARS-CoV-2. Antiviral activity of single compounds against SARS-CoV-2 as detected by qPCR in **a)** apical wash and **b)** intracellular lysate at 72 hpi. Top 16 compounds based on the viral content of apical wash at 72 hpi (n = 2–3, except for conditions tested at both investigatory sites: n = 5 for 10 µM nelfinavir, camostat; n = 6 for 30 µM molnupiravir; and n = 12 for 10 µM remdesivir and molnupiravir. HHT: homoharringtonine. Data are represented as mean ± standard deviation (SD). Dashed line: limit of what is considered significant antiviral activity in the current study (>1 log).

We evaluated the antiviral potential of 99 single compounds against apical SARS-CoV-2 infection in reconstituted nasal epithelium. Summarized data of all tested compounds can be found in Table S1 (n = 95, 4 compounds were excluded based on the loss of epithelial integrity). Of the 99 compounds tested, 16 exhibited > 1 log reduction in viral RNA in either apical wash (Fig. 2a) or intracellularly (Fig. 2b) at 72 hpi at the highest concentration tested. In general, the reduction of viral RNA was equal or even more pronounced when measured intracellularly compared to apical wash. Those compounds exhibiting strong inhibition of the replication of SARS-CoV-2 in this study primarily target individual parts of the viral replication machinery. Here, the most efficient inhibitor based on concentration alone was PF-00835231, a SARS-CoV-2 specific protease inhibitor, resulting in a 4–5 log reduction of viral RNA at 30, 10, (Fig. 2) and 5 µM (Fig. 4). Following closely are the RNA dependent RNA polymerase (RdRp) inhibitors remdesivir and molnupiravir, which also resulted in efficient reduction of SARS-CoV-2 replication at various concentrations. Remdesivir, included as positive control in all screening series, resulted in 3–4 log reduction in viral RNA in apical wash at 10, 5 (Fig. 2) and 1.15 µM (Fig. 4). Similar reduction (3.3 log) was observed after treatment with the orally active RdRp inhibitor, molnupiravir at 30 µM (Fig. 2a). Both remdesivir and molnupiravir were tested multiple times at two separate testing sites with minimal deviation, demonstrating the robustness of our infection protocol, detection methods and assay transferability (Fig. S2). Four other compounds, the HIV-specific protease inhibitors, nelfinavir and ritonavir as well as the protease inhibitors TO-195 and ONO-3307, resulted in an average 2–3 log reduction of SARS-CoV-2 RNA at the highest concentration tested (30 µM). Digitoxin, a cardiac glycoside, was also antiviral at a low concentration (0.36 µM) to similar levels as these non-specific protease inhibitors. Narasin, a coccidiostat and anti-bacterial also exhibited slight antiviral activity. Nafamostat, inhibitor of TMPRSS2 transmembrane serine protease, essential for SARS-CoV-2 host cell entry, resulted in average viral RNA reduction of 1.4 logs in apical wash at 30 µM, while the reduction in intracellular RNA was four times higher. Furthermore, in our experimental model, treatment with emetine, ivermectin, homoharringtonine (HHT), alisporivir, camostat, and mitoguazine resulted in borderline one log reduction of viral RNA in either apical wash or intracellularly at the highest concentration tested (Fig. 2).

### 3.3 Dose-response of molnupiravir treatment against SARS-CoV-2

**Fig. 3:**
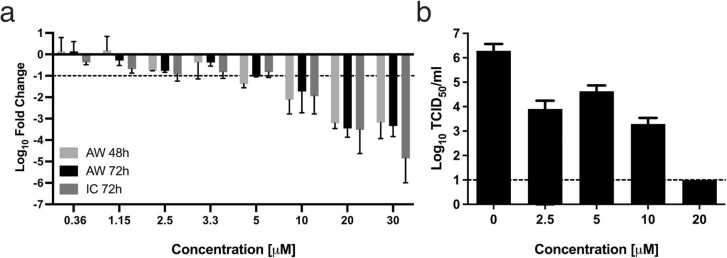
Dose-response of molnupiravir treatment against SARS-CoV-2 (a) Antiviral activity of molnupiravir at 48 and 72 hpi as detected by qPCR in apical wash. Data are represented as mean ± SEM, n = 6, 6, 2, 6, 2, 12, 4 and 6 for 0.36, 1.15, 2.5, 3.3, 5, 10, 20 and 30 µM, respectively. Dashed line: limit of what is considered significant antiviral activity in the current study (>1 log). (b) Remaining infectivity in apical wash after treatment with molnupiravir (TCID_50_/ml) (n = 2–4). Data are represented as mean ± standard deviation (SD). Dashed line: limit of detection (LOD).

The antiviral activity of molnupiravir against SARS-CoV-2 was tested at multiple concentrations and revealed antiviral activity (>1 log reduction at 72hpi) as determined by qRT-PCR at 10, 20, and 30 µM while some lower concentrations presented as borderline antiviral (Fig. 3a). Upon titration of apical secretions, no infectious virus was detected after treatment with 20 µM molnupiravir while 10, 5, and 2.5 resulted in 2–3 log reduction of infectious titer at 72 hpi (Fig. 3b). Based on these data, 10 µM was chosen as a baseline concentration for subsequent combination treatment.

### 3.4 Antiviral activity of molnupiravir-based combinations against SARS-CoV-2

**Fig. 4:**
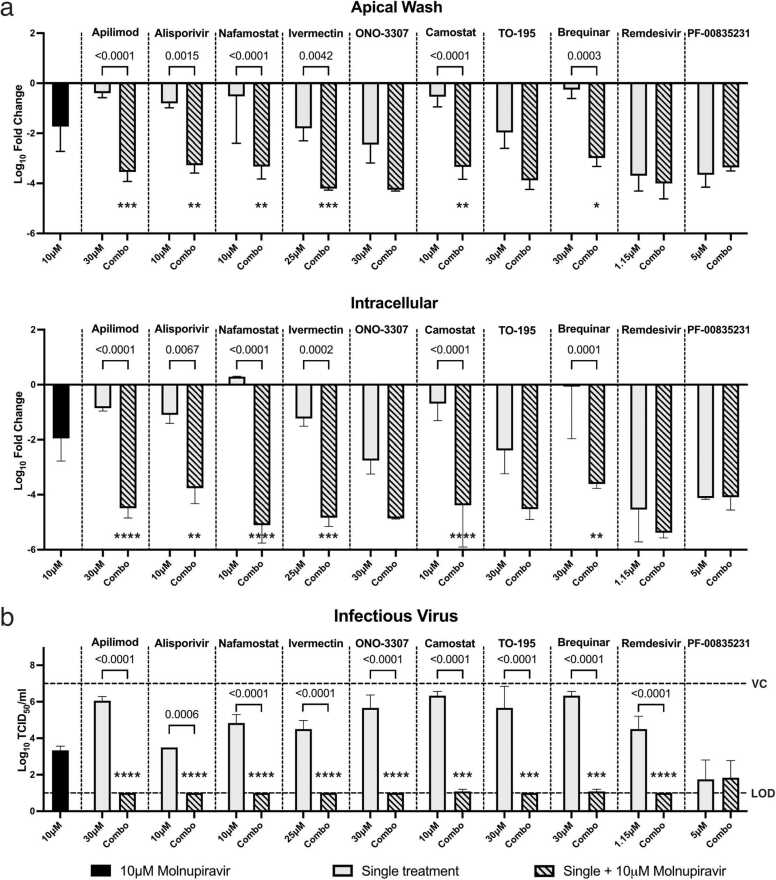
Antiviral activity of compound combinations against SARS-CoV-2. Antiviral activity of molnupiravir-based combinations against SARS-CoV-2 as determined by (a) qPCR in apical wash or intracellularly at 72 hpi (n = 2–4, and n = 12 for molnupiravir) and (b) TCID_50_/ml in apical wash at 72 hpi (n = 2–4). Dashed lines represent the limit of detection (LOD) and vehicle control (VC). Black column: 10 µM molnupiravir alone, gray columns: single compound treatments, striped columns: molnupiravir-based combinations. *p < 0.05, **p < 0.01, ***p < 0.001, **** p < 0.0001, representing significance over molnupiravir alone. Statistical significance between single agents and combinations are shown numerically. Data are represented as mean ± SD.

Overall, 30 combinations were tested at various concentrations (48 different conditions, Table S2). Treatment of SARS-CoV-2 infected epithelium with 10 µM molnupiravir alone resulted in a moderate 2 log reduction in viral RNA both in apical wash and intracellularly (Fig. 4a, black column). The combination of molnupiravir at a submaximal concentration with other compounds resulted in significantly higher antiviral activity measured by qPCR for apilimod, alisporivir, nafamostat, ivermectin, camostat and brequinar (Fig. 4a, striped bars). These compounds showed limited antiviral effect alone (Fig. 4a, gray bars) and the combined effect was also significantly better than treatment with molnupiravir alone (Fig. 4a, black bars). Interestingly, when looking at remaining infectious virus in apical wash, all combinations except molnupiravir and PF-00835231 showed significant reduction compared to single treatments, many conditions resulting in no detectable infectious virus (Fig. 4b). Combinations with ONO-3307, TO-195, and remdesivir, that did not exhibit any statistically significant benefit in the qPCR analysis, also presented with no detectable infectious virus at 72 hpi.

### 3.5 Secretions of IP-10 and RANTES after combination treatment

The secretion of two major chemokines induced by SARS-CoV-2 infection, IP-10/CXCL-10 and RANTES/CXCL-5, was moderate, with an average of 4.5 and 9.2-fold increase over mock respectively. In the combination treatments, we did not observe any robust pattern of inhibition. IP-10 secretion was generally low, and only significantly reduced in combination treatment with remdesivir (p < 0.0001). For nafamostat, ivermectin, ONO-3307, and brequinar no reduction was observed after the addition of molnupiravir (Fig. S3a) while a slight non-significant reduction was observed for alisporivir and TO-195. A similar pattern was observed for RANTES (Fig. S3b).

## 4 Discussion

Despite current access to vaccination against SARS-CoV-2, well-tolerated and effective antiviral treatment options should be pursued and refined for human use. For optimal antiviral treatment, a drug combination should interfere with virus replication and disease progression at multiple crucial levels to provide a comprehensive treatment against both the virus infection and the resulting disease. Furthermore, the risk of escape mutations that render virus-targeting drugs ineffective is lower with a multidrug treatment approach, as observed during treatment against HCV and in antiretroviral treatment against HIV [36], [37], [38]. In the current study, we have assessed the antiviral potential of compounds from the ReFrame and MMV drug libraries against SARS-CoV-2, both alone and in combination at two separate testing sites. We successfully transferred a standardized protocol using commercially available reconstituted airway epithelia, MucilAir™ [39], facilitating both reproducibility of results and a clear distinction between antiviral and toxic effects using trans-epithelial electrical resistance as a surrogate marker of toxicity, an important feature of this organotypic testing system [40]. Indeed, we observed increased toxicity beyond that of vehicle control after treatment with certain non-toxic compounds only after infection with SARS-CoV-2. This observation points towards the potential of synergistic cellular toxicity between certain drugs and SARS-CoV-2 infection, highlighting the need for extensive toxicity testing both prior to and during antiviral compound testing.

In the current study, the top five most active single treatments were all compounds that interfere with a variety of proteases or directly with the RdRp, *i.e*., PF-00835231, remdesivir, molnupiravir, nelfinavir, and ritonavir. Other protease inhibitors, ONO-3307 and TO-195 were also antiviral at the highest concentration tested (30 µM). PF-00835231 is the active metabolite of lufotrelvir, which is currently part of the pivotal ACTIV 3 clinical trial expected to report later in 2022. Other borderline antiviral compounds included digitoxin, narasin, nafamostat, and ivermectin with varying mechanisms of action (MoA). However, compounds with such borderline antiviral activity are likely ineffective clinically, as has been observed for ivermectin [41], [42], [43]. Interestingly, antimalarial compounds, including the 4-aminoquinolines chloroquine, amodiaquine, and pyronaridine, did not show antiviral activity in this assay, in contrast with results from other cell lines [44], [45]. It is not unusual to observe differences in the efficacy of antiviral drugs between *in vitro* and *in vivo* studies. Therefore, it is important to screen potential drug candidates in relevant cell culture systems to bridge this gap. Combination treatment based on the orally bioactive RdRp inhibitor molnupiravir at 10 µM, resulted in varying levels of viral inhibition with statistically significant improvement in antiviral activity for apilimod, alisporivir, nafamostat, ivermectin, camostat, and brequinar when evaluated by qPCR. Interestingly, all top 10 candidates, apart from PF-00835231, showed significant reduction in infectious titer in combination with molnupiravir compared to treatment with a single agent, highlighting the importance of comprehensive molecular and functional analysis of antiviral compounds. It should be mentioned that the concentration of PF-00835231 in the combination treatment results in significant reduction of virus replication alone, making it challenging to discern if combining it with molnupiravir would provide any benefit. Therefore, our future studies are focused on therapies with much lower concentrations of PF-00835231. Lastly, we observed only moderate upregulation of the chemokines IP-10 and RANTES after infection with SARS-CoV-2 and limited impact of combination treatments on their secretion. As a result, we were unable to discern any pattern of benefit.

In the current study, we have performed a medium-throughput screening of single antiviral compounds and compound combinations against SARS-CoV-2 in a physiologically relevant cell culture model. Furthermore, we have shown that our standardized infection and treatment protocol can be implemented at different test sites without much deviation, allowing for better comparison of drug efficacies between laboratories. This cell culture model provides an intermediate step between antiviral studies in cell lines and *in vivo* tests in humans and animals. Despite these advantages, this system is not without limitation. Although the epithelial layer contains various cell types, we can only elucidate the epithelial response to treatment and infection. Since the major driver of symptoms and severity in COVID-19 is not the pulmonary epithelium but rather the host immune system, future studies should focus on epithelial and immune cell co-cultures to clarify the interplay between infected pulmonary epithelium and the humoral immune response. Additionally, applying physiologically relevant concentrations of antiviral drugs in cell culture systems can be challenging. Here, we selected 10 µM of molnupiravir as a basis for combination treatment. This is likely on the lower end compared to doses seen to be effective in clinical trials. Furthermore, our protocol focuses on early treatment, which could be applied as a prophylactic treatment *in vivo*. As a result, we cannot discern or extrapolate the effectiveness of the compounds presented in the current study after the onset of symptoms or against severe disease.

In November 2021, Merck (MSD) and Ridgeback Biotherapeutics received emergency authorization from the American Food and Drug Administration for the use of molnupiravir against SARS-CoV-2 infection due to its modest efficacy in clinical trials [46]. When combined with Favipiravir, the antiviral activity of molnupiravir against SARS-CoV-2 in Syrian hamsters is enhanced [47], further highlighting the potential benefits of combination therapies. Molnupiravir is also orally bioactive, making its administration easy and straightforward. Furthermore, combination treatments could require lower dosages of single active compounds potentially providing additional therapeutic benefits.

## 5 Conclusion

The herein reported molnupiravir-based combination treatments increased antiviral activity against SARS-CoV-2 compared to single treatments in reconstituted nasal epithelium. The current experimental setup focuses on antiviral treatment one-hour post-infection. Therefore, treatment strategies using the compounds tested in the current study might be proposed as post-exposure prophylaxis. Further studies focusing on the precise additive or synergistic effects of molnupiravir-based combinations are needed to provide a more comprehensive idea of their treatment potential against SARS-CoV-2 *in vivo*.

## Funding

This work was funded by The 10.13039/100000865Bill and Melinda Gates Foundation, grant number INV-016776, and The Foreign, Commonwealth and Development Office (FCDO) of the United Kingdom. The funders did not have any role in the study design, data collection and analysis, decision to publish, or preparation of the manuscript.

## CRediT authorship contribution statement

Conceptualization, H.R.J., B.B., O.B.E. and S.C. Formal Analysis H.R.J., B.B., O.B.E. and S.C. Funding Acquisition T.W, S.C. Investigation, H.R.J., D.S., T.J., B.P., M.B., O.T., A.P. Methodology, H.R.J., B.B., O.B.E. and S.C. Project Administration M.R.-C., O.B.E., S.C. Resources K. S., T.W. Supervision M.R.-C., O.B.E., S.C. Validation H.R.J., D.S., B.B., O.B.E. and S.C Visualization, H.R.J. and B.B., Writing – Original Draft H.R.J., B.B., O.B.E. and S.C. Writing – Review & Editing, H.R.J., O.T., S.H., T.W., B.B., O.B.E., M.R.-C. and S.C.

## Declaration of interest

The authors have declared no conflict of interest.

## Data Availability

Data will be made available on request.

## Appendix A Supplementary material

.

Supplementary material Figure S1**: Determination of compound toxicity for concentration selection.** Typical representation of parameters determining compound toxicity; epithelial integrity (TEER), LDH release, ciliary beating frequency (CBF) on days 1, 2 and 3, for **a)** molnupiravir (n = 3) and **b)** LY2228820 (n = 2). Molnupiravir is well tolerated at both 30 and 10 µM while LY2228820 exhibits toxicity at 30µM: TEER is measured < 100 Ohm cm^-^^2^ at day 3, indicating a loss of tissue integrity, cytotoxicity on day 3 > 5%, which corresponds to a physiological turnover of the nasal epithelial model MucilAir™ and CBF was strongly decreased. Data are represented as mean ± standard error of the mean (SEM).

.

Supplementary material Figure S2**: Reproducibility of antiviral compound testing in MucilAir™ nasal epithelia between two test sites.** Repeated tests conducted with **a)** 5 µM remdesivir or **b)** 10 µM molnupiravir at two separate test locations using the same cell culture system and infection protocol. Data are represented as mean ± SEM, n = 2–3.

.

Supplementary material Figure S3**: Secretion of IP-10 and RANTES after combination treatment.** Secretion of the chemokines **a)** IP-10 (CXCL-10) and **b)** RANTES (CXCL-5) after molnupiravir-based combination treatment against SARS-CoV-2 as measured by ELISA (n = 2–4). White column: Mock (uninfected control), dark grey column: infected vehicle control containing 0.3% DMSO (VC), black column: 10 µM molnupiravir alone, light grey columns: single compound treatments, striped columns: molnupiravir-based combinations. *p < 0.05, * *p < 0.01, * **p < 0.001, * ** * p < 0.0001, representing significance over molnupiravir alone. Statistical significance between single agents and combinations are represented numerically. Data are represented as mean ± SD. n.e: not evaluated.
