# Supplementary material for: Molnupiravir combined with different repurposed drugs further inhibits SARS-CoV-2 infection in human nasal epithelium in vitro
Source: Biomed Pharmacother. 2022 Jun;150:None. doi: 10.1016/j.biopha.2022.113058 (PMC9057985; doi:10.1016/j.biopha.2022.113058)
Supplement: Supplementary file 2 — Supplementary material [file mmc2.docx]

**Table S2: Overview over all non-toxic compound combinations tested in the current study***.* Maximum tolerated concentration (≤30 µM) obtained during toxicity-testing and antiviral effects of different concentrations assessed at 48 and 72 hpi in apical wash and at 72 hpi intracellularly, data are represented as mean ± SD of log_10_ reduction over vehicle control (VC). MoA: Mechanism of Action, AW: Apical Wash, IC: Intracellular, SD: Standard Deviation

| Compound 1 | Compound 2 | MoA (1/2) | Conc.1  (µM) | Conc. 2 (µM) | AW 48h | (±)SD | AW 72h | (±)SD | IC 72h | (±)SD |
| --- | --- | --- | --- | --- | --- | --- | --- | --- | --- | --- |
| Molnupiravir | Alisporivir | RdRp/Cyclophilin | 10 | 10 | 2,728 | 0,486 | 3,732 | 0,286 | 4,292 | 0,110 |
| Molnupiravir | Amodiaquine | RdRp/Antimalarial | 10 | 10 | 2,407 | 0,154 | 2,343 | 0,587 | 3,034 | 0,107 |
| Molnupiravir | Apilimod | RdRp/PIKfyve | 10 | 30 | 2,613 | 0,032 | 3,560 | 0,431 | 4,517 | 0,419 |
| Molnupiravir | Baricitinib | RdRp/JAK | 10 | 30 | 1,926 | 0,206 | 1,327 | 0,051 | 1,734 | 0,769 |
|  |  |  | 10 | 10 | 1,379 | 0,223 | 0,940 | 0,112 | 1,022 | 0,054 |
| Molnupiravir | Brequinar | RdRp/DHODH | 10 | 30 | 2,278 | 0,123 | 2,986 | 0,339 | 3,609 | 0,161 |
| Molnupiravir | Camostat | RdRp/TMPRSS2 | 30 | 30 | 3,760 | 0,759 | 3,848 | 0,862 | 5,431 | 0,140 |
|  |  |  | 10 | 10 | 3,051 | 0,119 | 3,437 | 0,124 | 3,439 | 0,465 |
| Molnupiravir | Dexamethasone | RdRp/Glucocorticoid | 10 | 30 | 2,018 | 0,564 | 2,059 | 0,336 | 2,296 | 0,028 |
| Molnupiravir | Ebselen | RdRp/Antioxidant | 10 | 30 | 1,859 | 0,085 | 2,303 | 0,077 | 3,208 | 0,526 |
| Molnupiravir | Emetine | RdRp/Antiprotozoal | 10 | 1 | 1,844 | 0,898 | 2,783 | 1,056 | 3,886 | 0,858 |
| Molnupiravir | Fluvoxamine | RdRp/SSRI | 10 | 30 | 2,674 | 0,233 | 3,011 | 0,658 | 3,111 | 0,402 |
| Molnupiravir | Homoharringtonine | RdRp/Anti-tumor | 10 | 0,1 | 3,538 | 0,247 | 3,395 | 0,080 | 4,950 | 0,192 |
| Molnupiravir | IFN beta (IU/ml) | RdRp/Interferon | 30 | 200 | 3,132 | 0,374 | 3,966 | 0,421 | 4,145 | 0,839 |
|  |  |  | 10 | 200 | 2,575 | 0,419 | 2,874 | 0,116 | 3,066 | 0,535 |
|  |  |  | 10 | 100 | 2,621 | 0,140 | 2,878 | 0,318 | 2,634 | 0,736 |
| Molnupiravir | Ivermectin | RdRp/Antiparasitic | 30 | 30 | 3,225 | 0,143 | 3,885 | 0,119 | 5,349 | 0,272 |
|  |  |  | 10 | 25 | 2,618 | 0,051 | 4,236 | 0,063 | 4,882 | 0,321 |
|  |  |  | 10 | 10 | 3,031 | 0,366 | 2,783 | 0,346 | 3,096 | 0,164 |
| Molnupiravir | Mefloquine | RdRp/Antimalarial | 10 | 25 | 2,724 | 0,296 | 2,812 | 0,216 | 4,114 | 0,929 |
| Molnupiravir | Nafamostat | RdRp/TMPRSS2 | 30 | 30 | 3,891 | 0,346 | 3,420 | 0,804 | 5,718 | 0,773 |
|  |  |  | 10 | 30 | 2,979 | 0,230 | 4,284 | 0,270 | 4,542 | 0,151 |
|  |  |  | 10 | 10 | 3,394 | 0,765 | 3,391 | 0,090 | 5,113 | 0,783 |
| Molnupiravir | Nanchangmycin | RdRp/Tyrosine kinase | 30 | 1 | 3,524 | 0,006 | 3,489 | 0,805 | 5,260 | 0,224 |
|  |  |  | 10 | 0,3 | 3,512 | 0,368 | 3,072 | 0,027 | 1,719 | 0,336 |
| Molnupiravir | Narasin | RdRp/Antibiotic | 10 | 1 | 3,085 | 0,395 | 3,116 | 0,520 | 3,842 | 0,115 |
| Molnupiravir | Nelfinavir | RdRp/Protease | 30 | 30 | 4,132 | 0,214 | 3,494 | 0,133 | 4,633 | 0,234 |
|  |  |  | 10 | 10 | 2,932 | 0,408 | 3,182 | 0,047 | 3,810 | 0,740 |
| Molnupiravir | Niclosamide | RdRp/Multiple MOA | 20 | 10 | 3,190 | 0,180 | 2,812 | 0,661 | 3,236 | 0,368 |
|  |  |  | 10 | 10 | 2,520 | 0,398 | 3,900 | 0,253 | 4,230 | 0,430 |
| Molnupiravir | Nitaxozanide | RdRp/Multiple MOA | 20 | 10 | 3,774 | 0,329 | 3,498 | 0,019 | 4,398 | 0,011 |
|  |  |  | 10 | 30 | 3,012 | 0,066 | 3,265 | 0,110 | 3,806 | 0,094 |
| Molnupiravir | ONO-3307 | RdRp/Protease | 10 | 30 | 2,780 | 0,350 | 4,286 | 0,048 | 4,914 | 0,017 |
| Molnupiravir | PF-00835231 | RdRp/protease | 10 | 30 | 2,921 | 0,005 | 4,094 | 0,089 | 4,732 | 0,270 |
|  |  |  | 10 | 5 | 3,128 | 0,082 | 3,370 | 0,144 | 4,096 | 0,468 |
| Molnupiravir | Remdesivir | RdRp/RdRp | 10 | 1,15 | 3,040 | 0,031 | 4,000 | 0,620 | 5,379 | 0,192 |
|  |  |  | 10 | 0,36 | 2,322 | 0,948 | 2,775 | 0,416 | 3,086 | 0,790 |
| Molnupiravir | Ritonavir | RdRp/Protease | 10 | 10 | 2,619 | 0,567 | 3,148 | 0,054 | 3,515 | 0,398 |
| Molnupiravir | Samotilisib | RdRp/PI3K, mTOR | 10 | 1 | 0,939 | 0,295 | 1,580 | 0,317 | 1,476 | 0,247 |
|  |  |  | 10 | 3 | 2,546 | 0,077 | 1,817 | 0,187 | 1,591 | 0,132 |
| Molnupiravir | TO-195 | RdRp/Protease | 10 | 30 | 2,945 | 0,496 | 3,906 | 0,366 | 4,572 | 0,377 |
| Nelfinavir | Camostat | Protease/TMPRSS2 | 30 | 30 | 3,424 | 0,132 | 4,235 | 0,411 | 4,792 | 0,051 |
|  |  |  | 10 | 10 | 2,829 | 0,392 | 2,890 | 0,873 | 2,027 | 2,370 |
| Nelfinavir | Nafamostat | Protease/TMPRSS2 | 10 | 10 | 2,940 | 0,083 | 3,466 | 0,054 | 3,792 | 1,073 |
| Remdesivir | Nafamostat | RdRp/TMPRSS2 | 1,15 | 10 | 3,741 | 0,060 | 4,832 | 0,146 | 5,068 | 0,079 |
|  |  |  | 3,3 | 30 | 3,476 | 0,077 | 4,545 | 0,031 | 5,199 | 0,473 |
| Ritonavir | Camostat | HIV protease/TMPRSS2 | 30 | 10 | 2,854 | 0,216 | 3,448 | 0,440 | 3,459 | 0,275 |
