# Supplementary figures and images for: Molnupiravir combined with different repurposed drugs further inhibits SARS-CoV-2 infection in human nasal epithelium in vitro
Source: Biomed Pharmacother. 2022 Jun;150:None. doi: 10.1016/j.biopha.2022.113058 (PMC9057985; doi:10.1016/j.biopha.2022.113058)

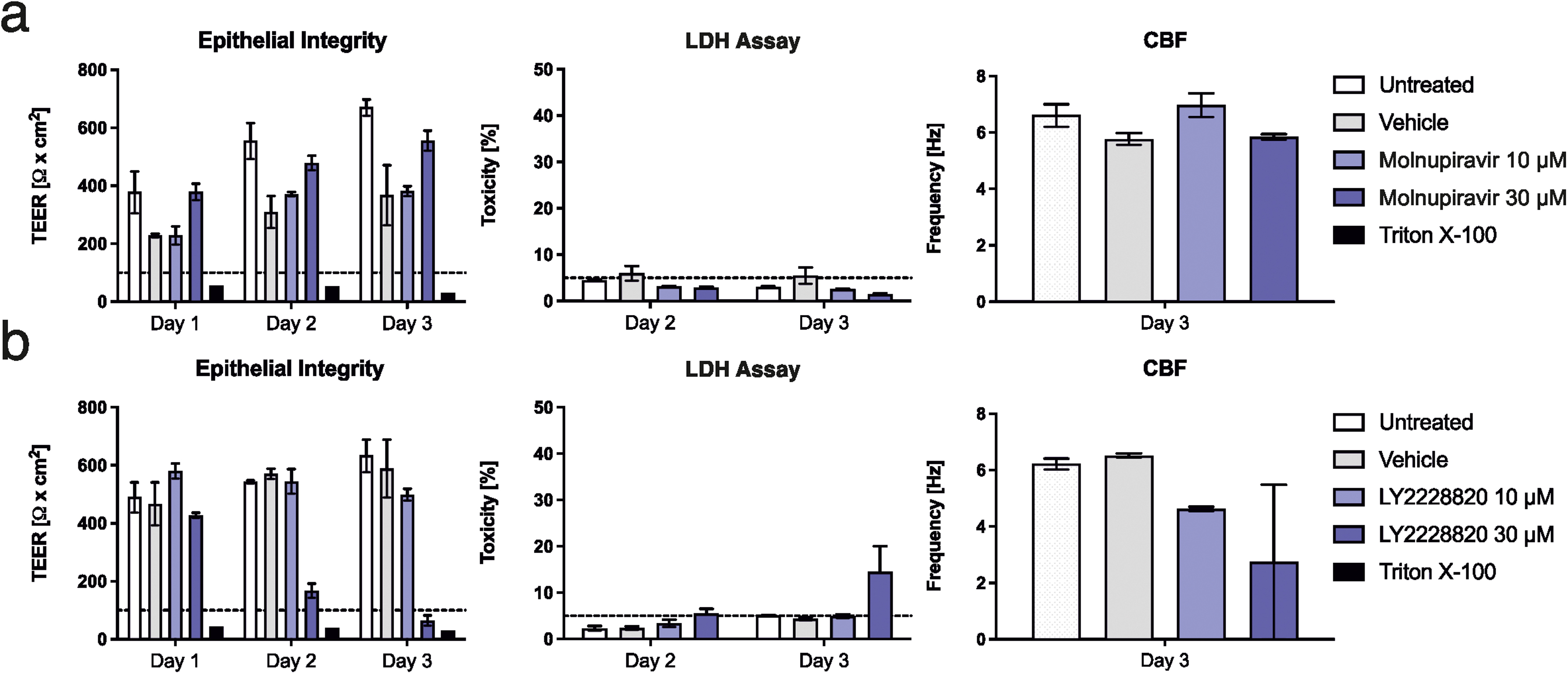

Supplement: Supplementary file 3 — Supplementary material Figure S1: Determination of compound toxicity for concentration selection. Typical representation of parameters determining compound toxicity; epithelial integrity (TEER), LDH release, ciliary beating frequency (CBF) on days 1, 2 and 3, for a) molnupiravir (n = 3) and b) LY2228820 (n = 2). Molnupiravir is well tolerated at both 30 and 10 µM while LY2228820 exhibits toxicity at 30µM: TEER is measured < 100 Ohm cm-2 at day 3, indicating a loss of tissue integrity, cytotoxicity on day 3 > 5%, which corresponds to a physiological turnover of the nasal epithelial model MucilAir™ and CBF was strongly decreased. Data are represented as mean ± standard error of the mean (SEM). [file mmc3.jpg]

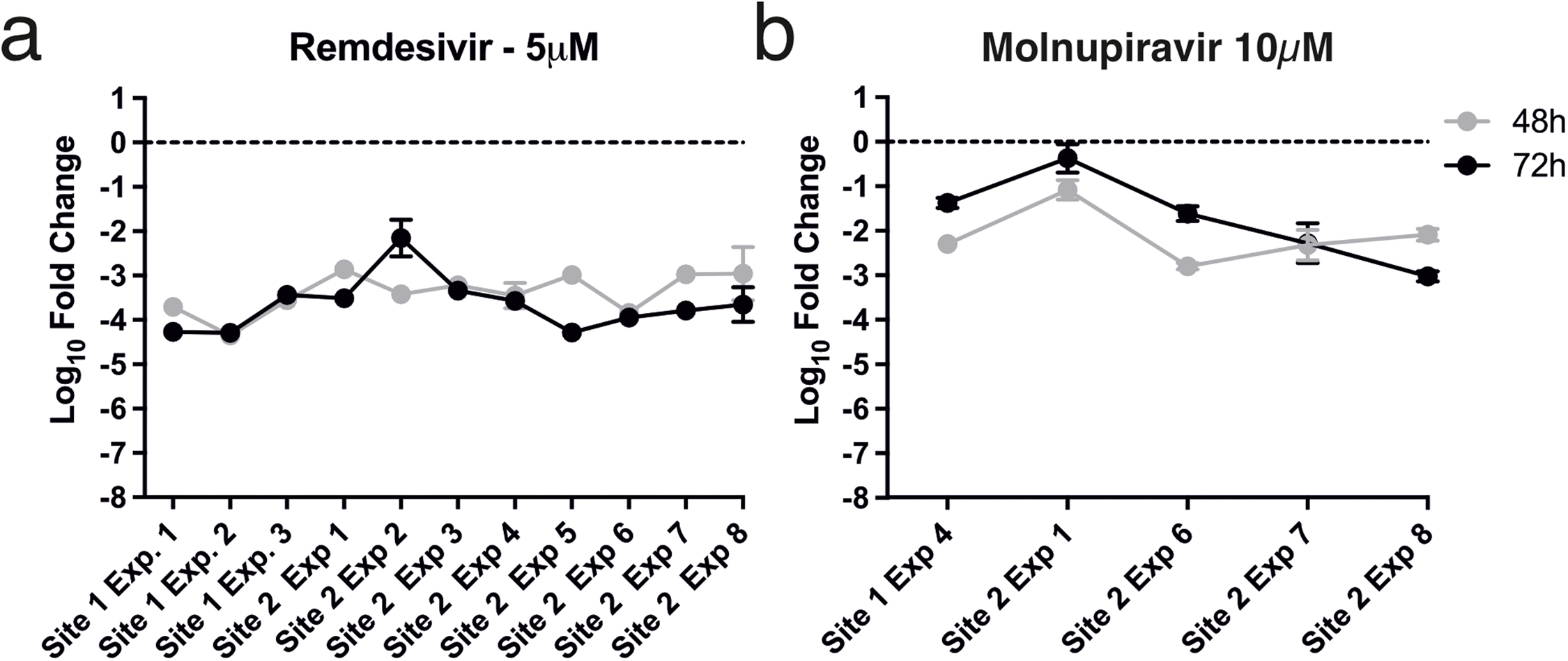

Supplement: Supplementary file 4 — Supplementary material Figure S2: Reproducibility of antiviral compound testing in MucilAir™ nasal epithelia between two test sites. Repeated tests conducted with a) 5 µM remdesivir or b) 10 µM molnupiravir at two separate test locations using the same cell culture system and infection protocol. Data are represented as mean ± SEM, n = 2–3. [file mmc4.jpg]

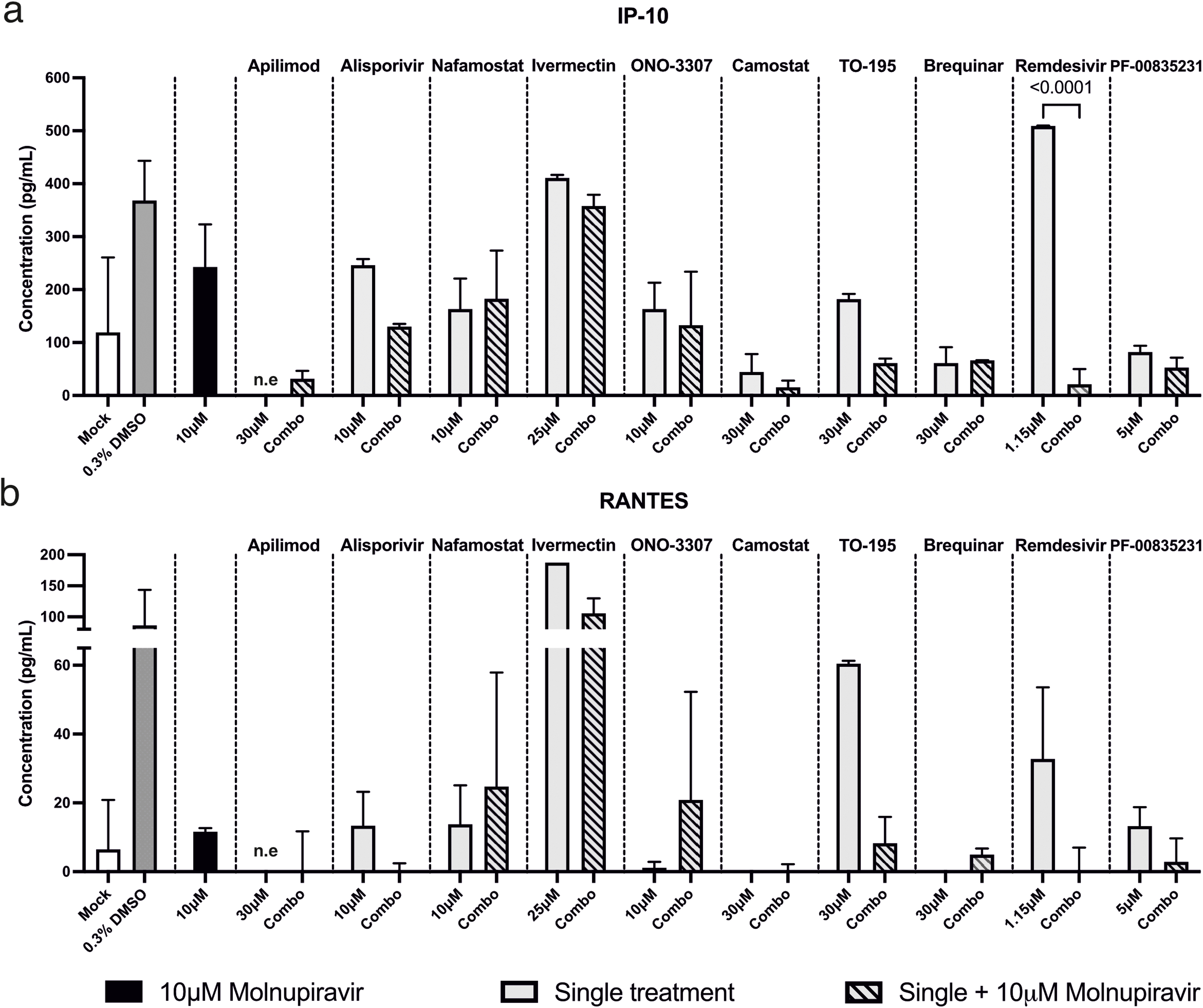

Supplement: Supplementary file 5 — Supplementary material Figure S3: Secretion of IP-10 and RANTES after combination treatment. Secretion of the chemokines a) IP-10 (CXCL-10) and b) RANTES (CXCL-5) after molnupiravir-based combination treatment against SARS-CoV-2 as measured by ELISA (n = 2–4). White column: Mock (uninfected control), dark grey column: infected vehicle control containing 0.3% DMSO (VC), black column: 10 µM molnupiravir alone, light grey columns: single compound treatments, striped columns: molnupiravir-based combinations. *p < 0.05, * *p < 0.01, * **p < 0.001, * ** * p < 0.0001, representing significance over molnupiravir alone. Statistical significance between single agents and combinations are represented numerically. Data are represented as mean ± SD. n.e: not evaluated. [file mmc5.jpg]
